# The Importance of the Gel Immersion Method to Successfully Identify the Jejunojejunal Anastomosis Site After Roux‐Y Reconstruction With Double‐Balloon Enteroscopy‐assisted Endoscopic Retrograde Cholangiopancreatography: A Case Report

**DOI:** 10.1002/deo2.70348

**Published:** 2026-05-10

**Authors:** Koki Nagano, Kohsaku Ohnishi, Yuki Ito, Naoko Hayata, Akino Okamoto, Takafumi Tanimoto, Shigeki Suemura, Motohiro Hirao, Atsushi Hosui, Naoki Hiramatsu

**Affiliations:** ^1^ Department of Gastroenterology and Hepatology Osaka Rosai Hospital Sakai Japan

**Keywords:** common bile duct stones, double‐balloon enteroscopy‐assisted ERCP, gel immersion method, jejunojejunal anastomosis, Roux‐en‐Y recsonstruction

## Abstract

The gel immersion method improves endoscopic visualization by displacing debris and blood with a viscous gel. Although it is commonly used in procedures such as endoscopic mucosal resection and endoscopic submucosal dissection, its use in double‐balloon enteroscopy‐assisted endoscopic retrograde cholangiopancreatography (DBE‐ERCP) has rarely been reported. An 81‐year‐old man with a history of distal gastrectomy and Roux‐en‐Y reconstruction underwent DBE‐ERCP for recurrent common bile duct stones. Despite 98 min of conventional attempts, the jejunojejunal anastomosis could not be identified because visualization was impaired by postoperative adhesions and residual food debris. After switching to the gel immersion method, the anastomosis was clearly identified within 4 min. Stone removal was successfully performed, and the postoperative course was uneventful. In this case, the gel immersion method markedly improved visualization and reduced intraluminal pressure, thereby facilitating identification of the anastomosis. This method may be particularly useful when conventional visualization is difficult during DBE‐ERCP, as it may enable rapid and safe identification of the jejunojejunal anastomosis.

## 1 Introduction

The gel immersion method, first reported in 2016, is a novel and increasingly utilized technique for improving visualization during gastrointestinal endoscopy [1]. Originally developed to assist endoscopic hemostasis in cases of active bleeding with poor visibility, this method is now also used in a wide range of diagnostic and therapeutic endoscopic procedures, including endoscopic submucosal dissection (ESD) [2], endoscopic mucosal resection (EMR) [3], and endoscopic ultrasonography (EUS) [4].

The principle of this method lies in its ability to create a stable and optically clear environment by replacing intraluminal gas or fluid with a viscous, transparent gel. The viscosity of the gel effectively displaces blood, bile, mucus, and debris from the endoscopic field. Unlike conventional water immersion, the higher viscosity of the gel minimizes turbulence and prevents rapid mixing with blood or secretions, allowing the visual field to remain clear for a longer period. This characteristic is particularly advantageous during therapeutic interventions in which even transient obscuration can compromise precision and safety.

In addition, the ability to perform endoscopic maneuvers under low intraluminal pressure reduces luminal distension and may improve patient comfort. The method may also improve scope maneuverability, especially in tortuous or surgically altered anatomy, and may reduce the risk of aspiration by minimizing the need for excessive insufflation and irrigation [5].

Double‐balloon enteroscopy‐assisted endoscopic retrograde cholangiopancreatography (DBE‐ERCP) is widely used for biliary interventions in patients with Roux‐en‐Y reconstruction. However, identification of the jejunojejunal anastomosis can be technically challenging due to adhesions, angulation, and poor visualization. While gel immersion has been reported to improve visualization during DBE in selected situations, its use for identifying the jejunojejunal anastomosis (Roux‐en‐Y anastomosis) has not been reported.

We report a case in which the gel immersion method enabled rapid identification of the jejunojejunal anastomosis during DBE‐ERCP after failure with conventional techniques.

## 2 Case Report

An 81‐year‐old man was admitted for the removal of recurrent common bile duct (CBD) stones. He had no subjective symptoms at admission. His medical history included branch‐duct intraductal papillary mucinous neoplasm of the pancreas, cholecystectomy, and partial colectomy with distal gastrectomy and Roux‐en‐Y reconstruction for transverse colon cancer with gastric invasion. Seven years earlier, he had undergone a cholecystectomy and DBE‐ERCP for the removal of CBD stones. The procedure had been technically challenging because of the marked angulation of the small bowel. The jejunojejunal anastomosis had been identified in 26 min, and the papilla had been reached in 38 min. Complete stone removal had been achieved. During withdrawal of the endoscope, tattoo marking of the jejunojejunal anastomosis was attempted due to difficulty maintaining endoscopic stability; two tattoos were placed on the blind‐end side relative to the anastomosis.

Three months before the current admission, he developed cholangitis due to CBD stones and underwent endoscopic biliary stenting with DBE‐ERCP. The jejunojejunal anastomosis had been identified in 13 min with the aid of the previous tattoo marks. The papilla had been reached within 20 min.

**FIGURE 1:**
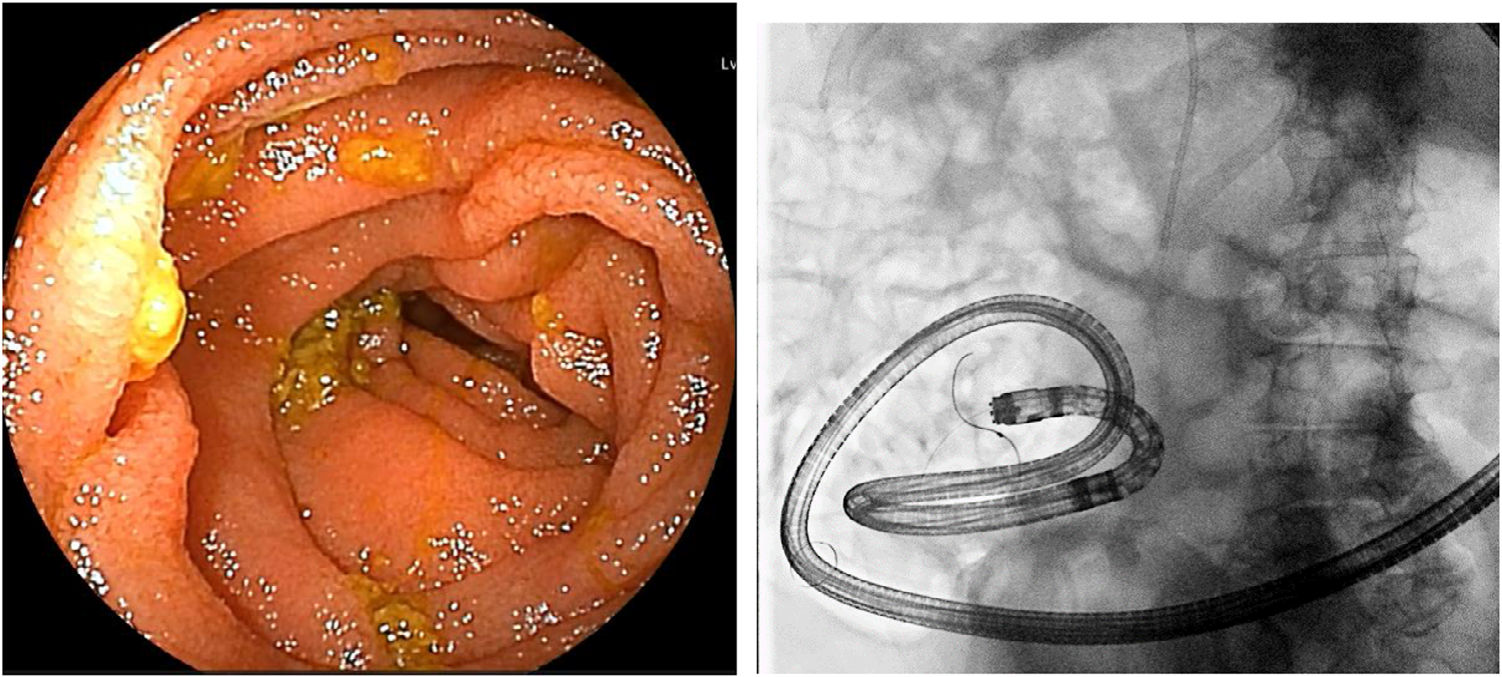
(A) Endoscopic image during double‐balloon enteroscopy‐assisted endoscopic retrograde cholangiopancreatography (DBE‐ERCP) showing difficulty in identifying the anastomosis. Because of residual food and strong peristaltic activity. (B) Fluoroscopic image while probing the lumen with a guidewire because of difficulty in identifying the anastomosis.

**FIGURE 2:**
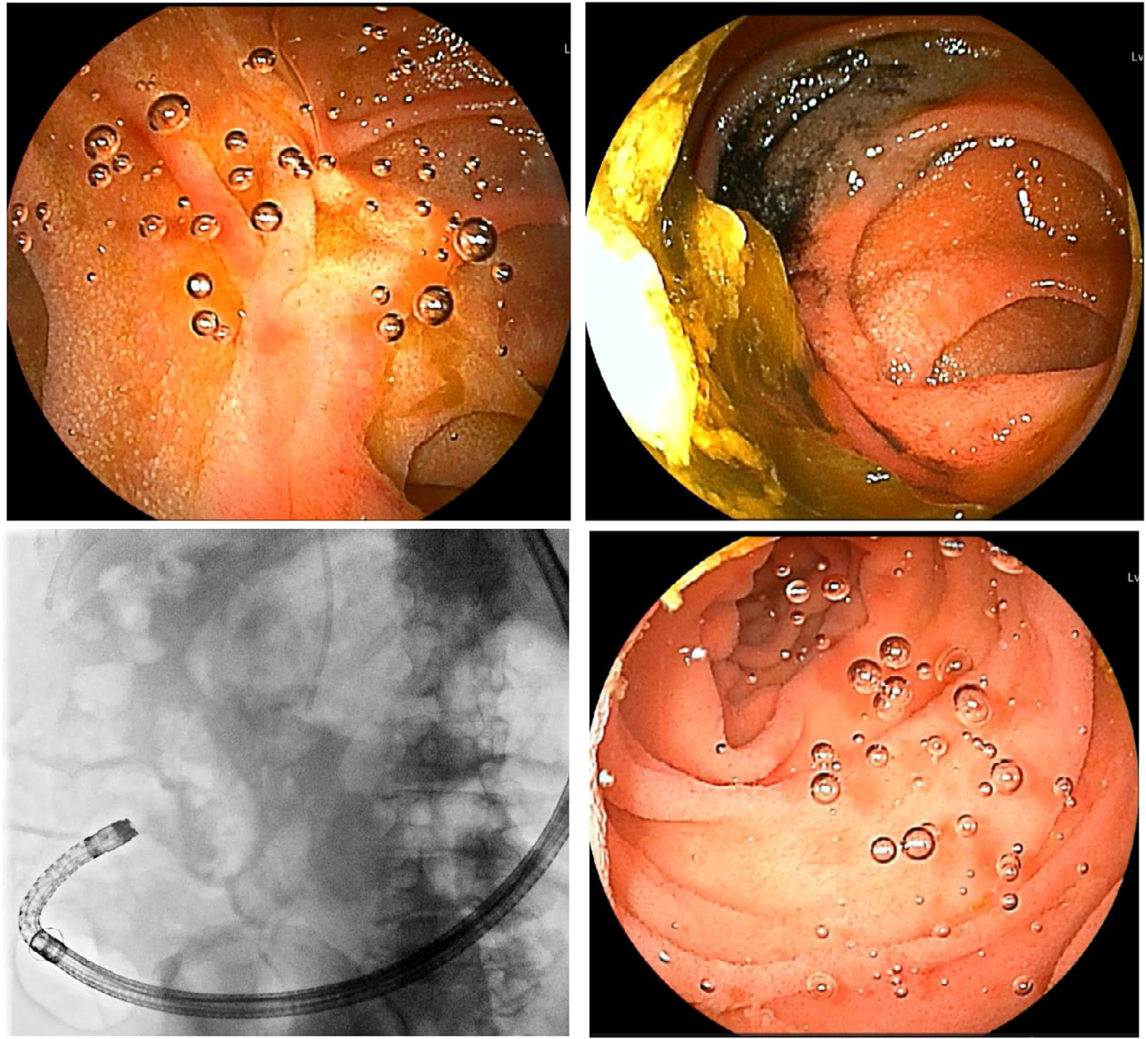
(A) Anastomosis clearly identified using the gel immersion method. (B) Endoscopic image of the anastomosis obscured by food residue after tattooing and insufflation. (C) Gel was injected after fluoroscopic confirmation that the scope had passed the anastomosis. (D) Endoscopic image showing a clear visual field with peristalsis suppressed.

He was subsequently readmitted for definitive stone removal. On admission, he was alert and afebrile. Physical examination revealed a soft, flat abdomen without tenderness. Laboratory findings were within normal limits. At the current admission, DBE‐ERCP was performed. However, adequate small bowel distension could not be achieved because of postoperative adhesions and residual food debris, resulting in poor visualization (Figure 1A). Despite 98 min of endoscopic searching, the jejunojejunal anastomosis could not be identified (Figure 1B). After switching to the gel immersion method, the anastomosis was successfully identified within 4 min (Figure 2A,B).

The gel immersion method was initiated from the jejunum distal to the anastomosis, as estimated fluoroscopically (Figure 2C), and the intestinal lumen was observed while the endoscope was gradually withdrawn (Figure 2D). First, the residual intraluminal gas was aspirated to collapse the intestinal lumen. Subsequently, the balloon at the distal end of the over‐tube was inflated, and refrigerated viscous gel (VISCOCLEAR, Otsuka Pharmaceutical Factory) was injected into the lumen using a syringe, as a water‐jet system was not available with the DBE. Although the injected gel typically disperses in all directions, inflation of the over‐tube balloon prevented proximal dispersion. This technique allowed the gel to expand preferentially on the distal side of the balloon, thereby creating a stable gel‐filled space ahead of the endoscope and suppressing backflow toward the oral side. Furthermore, balloon inflation prevented the inflow of intestinal fluid from the proximal side. This approach also allowed the procedure to be performed using only a small amount of gel, and finally, a total of two packages were sufficient.

**FIGURE 3:**
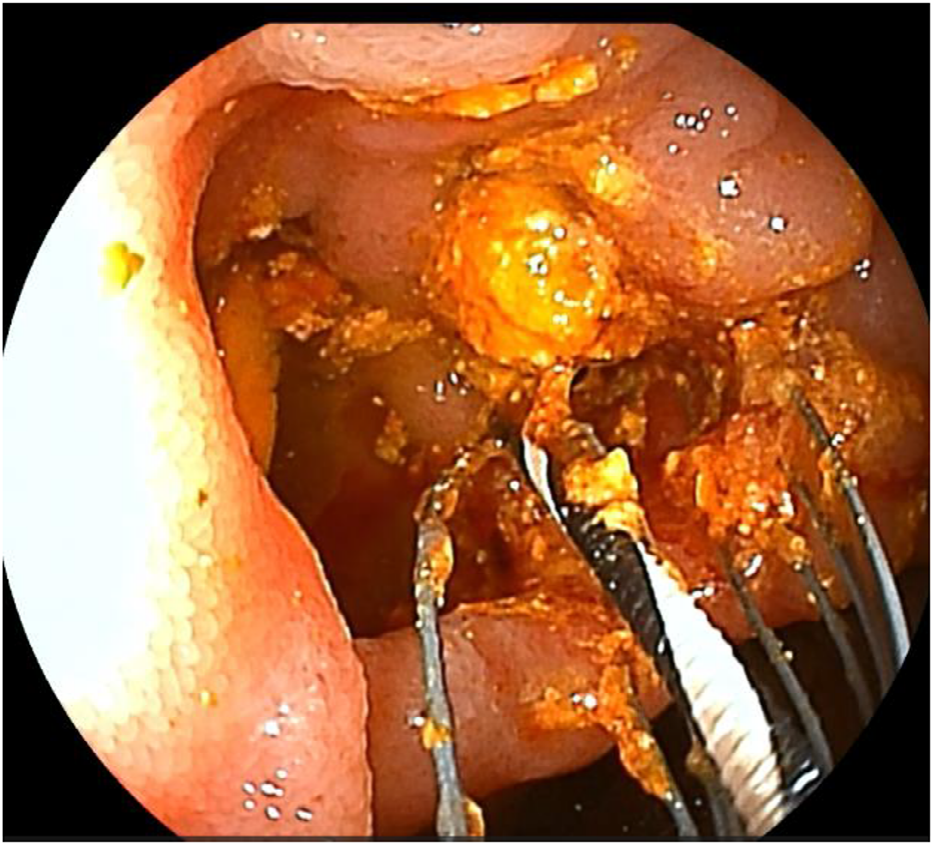
Endoscopic image removing the common bile duct stone.

After the papilla had been reached, cholangiography revealed multiple filling defects, suggesting the presence of CBD stones. Following endoscopic papillary large‐balloon dilation, the stones were removed using a basket catheter and a balloon catheter (Figure 3), and a nasobiliary drainage tube was placed. No postoperative complications, including pancreatitis, were observed. Cholangiography on postoperative day 6 confirmed complete stone clearance. The nasobiliary drainage tube was clamped on day 7 and removed on day 8. The patient was discharged on day 16 without complications.

## 3 Discussion

In DBE‐ERCP for patients with Roux‐en‐Y reconstruction, the average time required to reach the papilla has been reported as 48.9 min (range, 13–90 min) [6]. In cases involving distal gastrectomy with Roux‐en‐Y reconstruction, times ranging from 25.7 to 80 min have been reported [7]. In the previous session, the papilla had been reached within a reasonable time. Conversely, in the present case, identification of the anastomosis by conventional methods remained difficult even after 98 min, which substantially exceeded the previously reported range. Several factors contributed to the difficulty in this case. First, residual food debris impaired visualization. In the previous ERCP, the patient had undergone 3 days of fasting for cholangitis before the procedure. In the present admission, fasting was limited to the morning of the examination. This difference likely resulted in more residual food and poorer visualization. Second, as the procedure became prolonged, both visibility and scope maneuverability deteriorated further, making identification of the anastomosis increasingly difficult. Third, previous tattoos were located on the blind‐end side relative to the anastomosis and therefore did not enable identification of the anastomosis.

In contrast, the gel immersion method enabled rapid identification of the anastomosis within 4 min. This improvement can be explained by several mechanisms. First, aspiration of intraluminal gas followed by gel filling maintained a low intraluminal pressure, allowing visualization of areas that had previously been obscured by mucosal folds and angulations. Second, the use of refrigerated gel, with its relatively high viscosity, may have suppressed intestinal peristalsis. Third, the displacement of residual food particles created a clear field. Finally, evacuation of excess gas induced luminal collapse along the insertion route, reducing scope looping and improving the maneuverability.

A PubMed search with the terms “gel immersion” and “double balloon” found only 6 relevant reports. Some reports recommend switching from CO_2_ insufflation to the water or gel immersion method after entering the afferent limb, especially in cases with poor visualization due to bile or sludge [8]. Another report suggests that using the gel immersion method for searching the pancreaticoduodenal anastomosis in cases of stenosis after pancreaticoduodenectomy improves treatment outcomes [9]. However, to our knowledge, there have been no reports describing the use of gel immersion before entering the afferent limb, for the specific purpose of identifying the jejunojejunostomy as in the present case.

This report has several limitations. It describes a single case, and the effectiveness of the method may vary depending on patient anatomy and operator experience. Further studies are needed to evaluate the reproducibility and clinical impact of this technique in larger patient populations.

In conclusion, we report a case in which the gel immersion method proved effective for identifying the jejunojejunal anastomosis during DBE‐ERCP in a patient with Roux‐en‐Y reconstruction. Under the conventional method, visualization of the anastomosis was poor. However, the use of viscous gel provided a stable and clear endoscopic field, facilitated luminal observation, and enabled smoother scope advancement with a shorter procedure time. This case highlights the potential utility of the gel immersion technique in overcoming one of the major technical challenges in post‐surgical ERCP. The method may be useful not only for identifying biliary or pancreatic anastomoses but also for the jejunojejunal anastomosis in technically challenging DBE‐ERCP cases.

## Author Contributions

**Koki Nagano**: endoscopic procedures, clinical management (outpatient and inpatient care), writing – original draft, and writing – review and editing. **Kohsaku Ohnishi**: treatment planning and coordination, endoscopic procedures support, clinical management, and writing – review and editing. **Yuki Ito**: overall clinical supervision and writing – review and editing. **Naoko Hayata**: overall clinical supervision and writing – review and editing. **Akino Okamoto**: overall clinical supervision and writing – review and editing. **Takafumi Tanimoto**: clinical management, endoscopic procedures support, and writing – review and editing. **Shigeki Suemura**: overall clinical supervision and writing – review and editing. **Motohiro Hirao**: clinical management, endoscopic procedures support, and writing – review and editing. **Atsushi Hosui**: overall clinical supervision, final treatment decisions, writing – review and editing, and co‐guarantor of the entire case report. **Naoki Hiramatsu**: overall clinical supervision, writing – review and editing, and guarantor of the entire case report.

## Funding

The authors have nothing to report.

## Consent

Informed consent for publication, including endoscopic images, was obtained from the patient.

## Conflicts of Interest

The authors declare no conflicts of interest.
